# A Contemporary Insight Into the Seroepidemiology of Herpes Simplex Virus Infection in the Sub-Himalayan Region: Seroepidemiology of HSV Infection in North India

**DOI:** 10.1155/2025/6826627

**Published:** 2025-01-10

**Authors:** Sangeeta Deka, Mithilesh Kumar Jha, Pratima Gupta, Putul Mahanta, Deepjyoti Kalita

**Affiliations:** ^1^Department of Microbiology, All India Institute of Medical Sciences, Rishikesh, Uttarakhand, India; ^2^Department of Microbiology, Nagaon Medical College and Hospital (NMCH), Nagaon, Assam, India; ^3^Department of Microbiology, All India Institute of Medical Sciences, Patna, Bihar, India; ^4^Department of Microbiology, All India Institute of Medical Sciences, Deoghar, Jharkhand, India; ^5^Department of Forensic Medicine, Assam Medical College, Dibrugarh, Assam, India; ^6^Department of Microbiology, All India Institute of Medical Sciences, Guwahati, Assam, India

**Keywords:** herpes simplex virus, HSV-1, HSV-2, India, seroprevalence, Uttarakhand

## Abstract

Infection by human herpes simplex virus type 1 and type 2 (HSV-1/2) is common globally though with wide regional variability. Seroepidemiology of HSV-1/2 infections is of utmost importance in formulating control strategies, but there is a paucity of data from many regions of India. This study aimed to determine the prevalence of anti-HSV-1/2 antibodies in Uttarakhand and adjoining areas and to study its pattern and distribution in different subgroups. Serum samples from 322 cases were subjected to ELISA test to check for the presence of anti-HSV-1/2 IgG and IgM antibodies. Sociodemographic and clinical information were extracted from medical records. The association of seropositivity and associated factors was analyzed using Fisher's exact test. The overall HSV-1/2 seropositivity was observed to be 46.0% (95% confidence interval [CI]: 40.5–51.4). Total IgM and IgG were found in 6.2% (95% CI: 3.6–8.8) and 45.3% (95% CI: 38.1–48.9) cases, respectively. No significant difference between seropositivity of males and females was observed (45.7% in males versus 46.2% in females; *p* value: 0.928). Seroprevalence increased with age in both genders but was more pronounced in males (*p* value: <0.001), while 50.6% of women in the reproductive age group (18–30 years) were found to be seropositive. Females from Uttarakhand, compared to adjoining states (*p* value: 0.041) and both men and women residing in hilly terrain compared to plains (*p* value: 0.018; *p* value: 0.030), showed significantly lower prevalence, while urban-dwelling men showed higher seropositivity (*p* value: 0.048). Thus, HSV-1/2 seroprevalence is lower in this region, especially in young, rural, and hill dwellers, indicating majority are vulnerable to acquiring new infections. More awareness among high-risk groups and implementation of targeted public health policies can help control the disease burden.

## 1. Introduction

The human herpes simplex virus (HSV) includes two distinct types, that is, herpes simplex virus type 1 (HSV-1) and type 2 (HSV-2). Structurally, it possesses a double-stranded DNA and an envelope surrounding the virion. Human herpes simplex is one of the most common viral infections globally, with 60%–90% of adults harboring detectable antibodies. Globally, around 3.7 billion people aged less than 50 years (67%) and 491 million people between 15 and 49 years (13%) are estimated to be infected with HSV-1 and HSV-2, respectively [1–3]. It is maximum in African continent, South Eastern part of Asia, and West Pacific, followed by some regions of the American and European continents [1–3].

Herpes is characterized by lifelong infections and periodic reactivations due to various factors at the infection site [1, 2]. The transmission of HSV infection occurs by intimate, personal contact of a person harboring the virus with a seronegative person. HSV-1 transmission happens mainly by contact with sores in oral cavity, saliva, or skin in and around the mouth leading to orolabial or orofacial lesions while HSV-2 transmission primarily occurs via venereal route leading to genital herpes. However, increasing cases of HSV-1-related genital herpes have been reported in recent years [4, 5]. It is increasing to the extent of 20% of genital herpes cases indicating a significant role played by HSV-1 as sexual activity related disease [6]. Local symptoms in both primary and recurrent herpes include painful erythematous papules or vesicles, itching, soreness of throat/genitalia, and dysuria (in genital herpes). They are usually accompanied by systemic symptoms such as fever, malaise, and myalgia [1, 4, 7].

Depending on age, host immune status, and agent antigenic type, most herpes infections are without symptoms or with short-duration symptoms mostly going unnoticed [1, 4, 8]. So much that 75%–90% of genital herpes cases remain unaware about their infection primarily due to the absence of any visible signs and symptoms and thereby acting as silent spreaders [9]; however, in certain circumstances, herpes infection can lead to severe consequences. In immunocompromised state like in advanced HIV infection, it can cause frequent recurrences and severe and disseminated disease. HSV-1 can be linked to rarer consequences like encephalitis, keratoconjunctivitis, or other ocular sequelae. Pregnant women remain a high-risk population as an infant's prenatal or perinatal exposure to HSV-1 and HSV-2 (HSV-1/2) which can lead to neonatal HSV infection with severe fetal consequences [4, 7]. Therefore, knowledge of HSV's prevalence and determinants in a particular region, timely diagnosis, health awareness in the targeted population, and antenatal screening of pregnant women can be very effective in controlling the disease burden and preventing serious consequences. The frequency of HSV infection in a particular area is measured by testing for anti-HSV-1/2 antibodies by indirect serological tests, as both virus and the immune response are thought to persist during the entire life period of the host post infection.

The mean HSV-1 seroprevalence (pooled) in Asia was reported to be 50% in children and 76.5% for adults, with wide variations among countries [3, 6]. HSV-1 seroprevalence indicated a trend of lower prevalence in younger cohorts, thus increasing the risk of its acquisition by sexual route causing genital herpes [6]. This enhances the chances of congenital herpes. Khadr et al. reported a pooled mean prevalence of 67% in India [6]. However, considering India's geographical and demographic diversity, this region has extremely scarce data. Few studies, which estimated the burden of overall herpes infection in India, reported wide variability in seroepidemiology [10, 11].

Moreover, few studies reported low prevalence in subjects from Indian subcontinent which could not be explained easily [5, 12, 13]. Globally, females are at higher risk of acquiring HSV-1/2, more so in genital herpes [1–3, 8]. However, no differences in prevalence by gender are noticed in the Asian region [6].

Given this situation, we undertook this study to assess the gender-based seropositivity of HSV1/2 in our hilly state of province in Northern India, that is, Uttarakhand (with subjects hailing from adjacent areas too). We planned to evaluate the pattern and distribution of herpes infection in our subjects.

## 2. Materials and Methods

### 2.1. Setting

Study was conducted enrolling hospital-based subjects attending diagnostic labs under Microbiology Department of All India Institute of Medical Sciences, Rishikesh, India. In this cross-sectional study, subjects attending our laboratory between May 2015 and December 2019 were selected. Our Institute's hospital is a tertiary care teaching hospital and caters for the people of our province and adjoining areas of neighboring provinces of India. Uttarakhand province lies in the northern most part of India and consists of both river valley plains and mountain ranges of the great Himalayas. Subjects from the adjoining provinces such as western parts of Uttar Pradesh, Haryana, Delhi NCR (mostly plains), and Himachal Pradesh (Himalayan range) were also considered for the study. The institutional review board reviewed and approved our study protocol (AIIMS/IEC/2220/835–12.12.20). Patient confidentiality was maintained by assigning specific identifier number in lieu of name and registration number.

### 2.2. Inclusion and Exclusion Criteria

Samples were received from multiple departments and collected from patients with clinical suspicion of herpes as well as from those undergoing routine screening (antenatal, pediatric, etc.). Sociodemographic data and clinical history were extracted from the hospital information system and relevant medical records. Cases with the absence of appropriate data were excluded. Exclusion was also strictly adhered to in samples without label or scanty or hemolyzed, or lipemic, or/and leaking.

### 2.3. Sample Size

Formula *n* = *Z*^2^*P*(1 − *P*)/*d*^2^ (sample size (*n*); confidence level at 95%, that is, *Z* [taking standard value of 1.96]; expected prevalence of the disease in the particular area (*P*); precision (*d*) were the variables) [14]. Due to lack of published data on national and regional prevalence of HSV-1/2 (in India), the global baseline seroprevalence of 66.7% was considered (*p*=0.67) [1–3]. Considering 6% (*d* = 0.06) absolute precision, the sample size was calculated to be 306. Final sample size of 321 was fixed (5% increment from the calculated value) to account for the nonresponders.

### 2.4. Laboratory Methods

From each subjects, about 5 mL venous blood was collected, and serum was separated by centrifugation. Subsequently enzyme-linked immunosorbent assay (ELISA) was performed for detection of anti-HSV-1/2 antibody. IgM and IgG levels for HSV-1/2 were measured using Calbiotech Inc. (CBI) HSV 1 & 2 IgM and IgG ELISA kits (Calbiotech R5EC 96 well ELISA, California, USA) using the manufacturer's kit insert. Diluted patient serum was added to ELISA plate wells which are precoated with purified antigens. IgM-/IgG-specific antibody from serum will combine with these antigens (when present in serum). Kit's enzyme–coenzyme conjugate was then poured to bind to the antigen–antibody complex. After adding the substrate, it was hydrolyzed by the complex to give colorimetric change. The intensity of color in the assay increases proportionally with the quantity of IgM-/IgG-specific antibodies in the sample. For ELISA testing, we utilized a fully automated 7 plate system with robotic arm (Euroimmun Analyzer I-Walkaway automated seven plate ELISA reader, Euroimmun, Germany), with 450 nm optimal density (OD) reading. Interpretation was based on calculation of antibody index (AI) which was derived by dividing individual sample OD by cut off value (calibrator OD multiplied by calibrator factor). Calculated cutoff value of AI for positives was >1.1, and cutoff value for negatives was <0.9. An AI value between 0.9–1.1 was considered equivocal and retested by the same kit. If retested result was again equivocal, sample was excluded.

### 2.5. Statistical Analysis

All data were entered in Microsoft Excel spreadsheet and checked twice for errors. Graphs and tables were prepared as per spreadsheet function. A map depicting the study area was created using QGIS v3.24. Statistical analyses were done using standard statistical software (SPSS v23). Frequencies of the independent variable, mean, standard deviation (SD) and 95% confidence interval (CI) were calculated for the dependent variable. The relation of seropositivity with sociodemographic and relevant other factors was checked by applying Fisher's exact test. A two-sided *p* value of <0.05 was considered significant.

## 3. Results

### 3.1. Population Characteristics

Altogether, 322 cases were recruited into the study, out of which 151 (46.9%) were males and 171 (53.1%) females. Median age of the subjects was 20 years (S.D ± 6.0) and ranged from 1 day after birth (1 day–1 year taken as infants) to 71 years. The sociodemographic variables are listed in Table 1. About 17.7% of subjects were enrolled in 2017, while 39.4% and 42.9% cases were enrolled in 2018 and 2019, respectively. Almost three-fourths of the enrolled subjects practiced Hinduism, 21.7% Muslims and while 3.7% belonged to other faiths (Christian and Sikh). Most subjects (65.2%) hailed from Uttarakhand, while 30.4% came from Western Uttar Pradesh (UP) and 4.3% from other states. Around one-third of the subjects had dwellings in Uttarakhand's hilly terrains and adjoining areas. Most of the enrolled cases were referred from antenatal screening (37.3%) in Obstetrics and Gynecology Department and fetal screening (30.7%) from NICU and pediatrics department (Table 1).

### 3.2. Seropositivity

The seropositivity rate of HSV-1/2 was found to be 46.0% (*n* = 148) (95% CI: 40.5–51.4). Total IgM and IgG positivity was 6.2% (*n* = 20) and 45.3% (*n* = 140), with a mean AI of 0.37 (SD ± 0.47) and 1.39 (SD ± 2.17), respectively. The gender-wise prevalence of HSV type-specific antibodies is shown in Table 1. No significant difference was noted in overall HSV-1/2 as well as individual IgM and IgG seropositivity between males and females in the general population (*p* value: 0.928, 0.453 and 0.938, respectively).

### 3.3. Factors Associated

**Figure 1:**
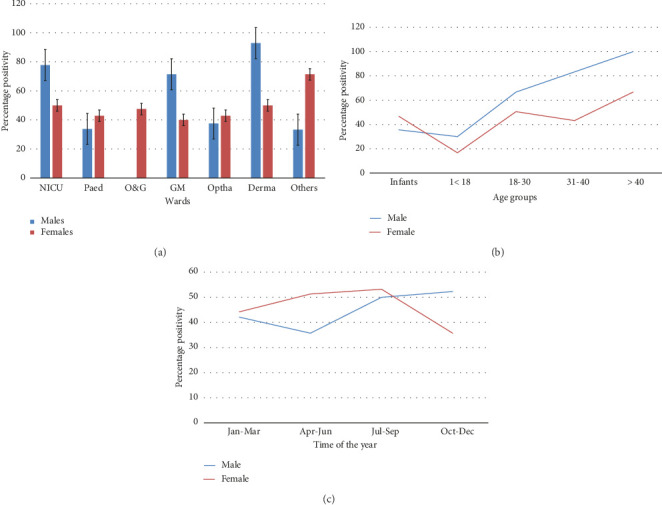
Gender-wise relationship of HSV seropositivity trends with respect to (a) different wards from which cases were referred, (b) different age categories, and (c) time of the year.

The gender-wise relationship between HSV-1/2 antibody prevalence and socioeconomic and other factors, as analyzed by Fisher's exact test, is presented in Table 2. An increasing trend of positivity with age was observed in both males and females (*p* value: < 0.005 and 0.050, respectively), but this trend was more pronounced in males with higher prevalence as age advances (Table 2 and Figure 1(b)). Only two of the 78 infants enrolled (2.6%, both males) showed the presence of IgM. Although seropositivity increased with increasing age, a slight peak was notices in infants of both genders due to higher prevalence of IgG. In females, the positivity rate was found to be higher in the hot and rainy months of July to September compared to the colder months (53.2% in July–September vs. 35.7% in October–December and 44.2% in January–March) (Figure 1(c)), but the association was statistically insignificant. Furthermore, no significant association between HSV1/2 seropositivity and any religion was observed though positivity in both the genders was higher in Muslims (Table 2).

Females from Uttarakhand exhibited a significantly lower prevalence of HSV-1/2 (38.9%) compared to the adjacent states (58.9% in west UP and 57.1% in other adjacent states) (*p* value: 0.041). Both male and female subjects who belonged to hilly areas were also at lower risk of acquiring infections compared to those living in plains (males: 32.7% vs. 53.5%, *p* value: 0.018; females: 33.3% vs. 51.7%, *p* value: 0.030). A borderline increase in HSV-1/2 seropositivity in men dwelling in urban areas compared to those in rural areas was observed (55.2% vs. 38%; *p* value: 0.048) while no significant rural–urban discrepancy was recorded in the case of females (50.6% in urban versus 41.5% in rural; *p* value: 0.149). Significantly higher seropositivity was noticed in male neonates from NICU (77.8%) and male subjects referred from general medicine (71.4%) and dermatology (92.9%) departments (Table 2) (Figure 1(a)).

## 4. Discussion

As per our search in different databases, current study was the first on HSV seroepidemiology from this sub-Himalayan region with a conglomeration of both river valley plains and hilly terrain. Lack of published data on HSV prevalence from this region in different databases prompted us to undertake this study. Overall, we found the seropositivity of HSV-1/2 to be lower than global baseline seroprevalence of 66.6% [1–3]. Contrary to high seroprevalence rates observed elsewhere, viz. ∼90% adults in Africa [3, 15], 73%–75% in Eastern Mediterranean and Western Pacific, ∼70% of women in the USA [16], and 76.5% of adult Asians [6]; we found the seropositivity of 46% for the overall study population. Seroepidemiologic studies on HSV-1/2 prevalence from the Indian subcontinent are scarce. Kaur et al. reported a high overall seroprevalence of 63% in 16–40 years adults attending family planning outpatient departments in Delhi [11]. Shivaswamy et al. also reported a very high HSV-1/-2 prevalence (82.9) in adults with or without high-risk behavior [17]. However, in concordance with our findings, Dinkar et al. found HSV prevalence to be 52% in clinically suspected patients and routine screening in high-risk groups [10]. Another study also found HSV-1/-2 prevalence to be much lower in Indian men compared to Filipino men living in Qatar [12]. Two studies from South India also reported low HSV-2 seroprevalence of 5.9% (in 15-49-year-old adults) and 6.7% (in pregnant women) [18, 19]. The climatic condition of this region and behavioral/cultural habits of the residents might have an impact on the low prevalence, which needs to be further explored with more extensive population-based studies.

Globally, females were more infected than males [1–3, 8, 18, 20]. In our study, although seropositivity in females was marginally higher than in males (46.2% vs. 45.7%), this difference was not statistically significant (Table 3). In tune with our finding, Khadr et al. also reported no differences in seroprevalence by gender in Asian countries [6]. We further did gender-based analysis of the data to check and compare the pattern as well as distribution of infection in both genders.

The presence of anti-HSV-1/2 IgG and IgM in the blood indicates past and current ongoing infection. A rise in only IgM indicates actual primary infection, and both IgM and IgG indicate nonprimary recurrent infection, while elevated IgG levels indicate past infections. Therefore, most studies reported a common trend of increasing infections both HSV-1 and HSV-2 by age, mirroring the cumulative effect of infections acquired over the ages [2, 6, 10, 11, 20, 21]. As expected, we too observed higher prevalence of HSV-1/-2 with increasing age in both genders. However, this trend was more prominent in the males (*p* value: <0.001 vs. 0.050), with all the 11 male subjects > 40 years showing antibodies against HSV-1/-2. Similar observations were made by Looker et al., who reported higher seroprevalence in the oldest age group of males [3]. In females, although an increase with age trend was noticed, a comparatively high prevalence (50.6%) was noticed in the 18–30 years age bracket, probably due to associated sexual behavior in that set of population. Also, the seroprevalence of females in children (1–18 years) was much lower than males (16.7% vs. 30.0%), making them more vulnerable to new infections. Interestingly, Nasrallah et al. observed that this trend of increasing seroprevalence with age was significant in Indian males but not in Filipino males [12].

In our study, higher seropositivity was observed among Muslims than among other religions. It was more pronounced in the males (66.7% in Muslims compared to 42.5% in Hindus and 28.6% in other religions) than in the females (56.5% in Muslims compared to 42.5% in Hindus and 40% in other religions). However, the association was not statistically significant (marginally insignificant in males with a *p* value of 0.061) (Table 2). Effects of residual confounding factors such as socioeconomic factors, living conditions, and cultural behaviors leading to this association cannot be ruled out. However, in some other studies, HSV-2 and circumcision were found to be positively associated [18, 22]. More extensive population-based studies exploring other factors are needed to draw a conclusion.

Studies on HSV-1/-2 seroepidemiology depict considerable variation geographic location-wise, including reasonable variations within even regions [1–3]. Jennings et al. found wide differences in the prevalence of HSV-2, ranging from 4.2% to 49.1% in Tamil Nadu, India, based on a study [23]. Similar regional variations were also observed in studies conducted elsewhere [21, 24, 25]. Our current study also recorded a geographical variation of HSV-1/2 infection. Females from the state of Uttarakhand had a significantly less chance of having HSV-1/2 infection compared to females from adjoining states (*p* value: 0.041). Low population density, climatic conditions, and cultural habits might have a role in lower prevalence.

Another striking finding of our study is that both males and females residing in hilly terrain had significantly lower prevalence. Moreover, we observed relatively higher HSV-1/-2 seropositivity compared to rural men in urban dwellers. This difference in prevalence (rural vs. urban and hills vs. plains) may be due to lower population density in hills and villages. The study by Bunzli et al. points out that towns with residents larger than 1500 inhabitants had higher seroprevalence compared to less than 1500 inhabitants [25]. Moreover, the likelihood of these findings may be due to residual confounding with sociocultural and behavioral factors of the local inhabitants cannot be ruled out, and these were not explored in this study. Other studies conducted elsewhere also corroborate our finding of higher prevalence in urban dwellers [25–28]. However, few studies documented either no rural–urban discrepancy or a rural preponderance [20].

The spectrum of clinical presentations in the symptomatic cases varied among males and females. More male neonates referred from the NICU showed antibodies against HSV-1/-2 (77.8%). Male cases referred from general medicine and dermatology departments also showed higher seropositivity. Anti-HSV antibodies in the majority of the female patients were detected during routine antenatal screening. The early detection and treatment of symptomatic genital herpes reduce the HIV infection rate as there is evidence of increased transmission of HIV in herpes patients with genital ulcers [29], and maximum transmission is seen in the same age group for both diseases. We should be cautious in immunizing HSV seropositive cases with the influenza vaccine, and HSV-1 central nervous system reactivation can happen following influenza vaccination [30].

A large number of seronegative populations, as observed in our study (overall 54%), especially younger females of less than 18 years (83.3% seronegative), are a matter of concern as they are highly vulnerable to herpes infection when they reach the reproductive age group. Korr et al. observed that reduced seroprevalence of HSV-1 and HSV-2 potentially makes more people susceptible to HSV-1/-2-mediated genital infections [4]. Moreover, we noticed increased prevalence in 18-30-year-old females (50.6%), mirroring the fact that most females acquired new infections in the reproductive age. This is alarming as an infection acquired during pregnancy leads to congenital herpes, which in turn results in serious fetal morbidity and mortality. This mandates routine antenatal screening in this region's pregnant women (both IgM and IgG). HSV-1/-2 antibody avidity test can also help assess the risk of vertical transmission. Significant decline in HSV-1/-2 seroprevalence over time had been reported in studies from the USA, Germany, and other European states due to improved hygiene and awareness [4, 6, 30, 31]. Thus, more youths reach the sexually active phase of life prior to nonsexual exposure to HSV-1 and are at a higher risk of acquiring the infection genitally, thereby increasing the chances of congenital herpes. The past 2 decades saw a substantial spike in genital herpes cases related to HSV-1 [2–5, 30, 32].

The limitation of current work (being a hospital-based work) includes the inability to analyze a few putative risk factors such as household conditions, sexual behavior, and cultural practices. Also being a serology-based study genital HSV infections and oral HSV infections could not be distinguished.

## 5. Conclusion

To conclude, HSV-1/2 seropositivity is lower in this region relative to baseline global seroprevalence, and unlike female preponderance worldwide, this study did not find any gender predilection for herpes infection. Younger persons, the hill population, and females from Uttarakhand have relatively low disease loads. In contrast, urban males, male neonates, and females in the reproductive age group were found to be more vulnerable to herpes infections. These findings call for awareness and early diagnosis in the targeted population and an unmet need for effective control of neonatal herpes and the well-being of women of childbearing age.

## Figures and Tables

**Table 1:** Characteristics of the study population.

Characteristics		No. of cases, *n*	Percentage %
Age categories^1^	Infants	78	24.2
	< 18	58	18.0
	18–30	114	35.4
	31–40	49	15.2
	> 40	23	7.1

Gender	Male	151	46.9
	Female	171	53.1

Year	2017	57	17.7
	2018	127	39.4
	2019	138	42.9

Time of year	Jan–Mar	62	19.3
	Apr–Jun	81	25.2
	Jul–Sep	93	28.9
	Oct–Dec	86	26.7

Religion	Hindu	240	74.5
	Muslim	70	21.7
	Others	12	3.7

States	Uttarakhand	210	65.2
	West UP2	98	30.4
	Others^3^	14	4.3
	Hills^4^	106	32.9
	Plains^5^	216	67.1
	Rural	166	51.6
	Urban	156	48.4

Wards	NICU	11	3.4
	Paed	88	27.3
	O&G	120	37.3
	GM	26	8.1
	Optha	28	8.7
	Derma	18	5.6
	Others	31	9.6

^1^For age categories, the lower limit is the completed given age (in years) and the higher limit is completed given age (in years) up to one day less than the succeeding year.

^2^West UP includes Bijnor, Najibabad, Muzzafarnagar, Shaharanpur, Kashipur, Deoband, Khatauli, Rehmatganj, Sangipur, Sewarampur, Moradabad.

^3^Other states include nearby areas of Himachal Pradesh, Haryana, Delhi NCR.

^4^Hills include Tehri-Garhwal, Pauri-Garhwal, Kumaun ranges, Uttarkashi, Narendra nagar, Rudraprayag, Mussoorie, parts of Dehradun, Srinagar.

^5^Plains include Rishikesh, Haridwar, Roorkee, Jwalapur, parts of Dehradun and Ranipokhari, parts of Kotdwar, Raiwala, and Rampur.

**Table 2:** Gender-wise association between variables and herpes simplex virus 1 and 2 seropositivity as tested by Fisher's exact test.

Characteristics	Males (*N* = 151)				Females (*N* = 171)			
	Total *N* (%)^2^	Pos *n* (%)^3^	Neg *n* (%)^3^	Exact sig. 4	Total *N* (%)^2^	Pos *n* (%)^3^	Neg *n* (%)^3^	Exact sig. 4
Age category1								
Infants	73 (48.3)	26 (35.6)	47 (64.4)	**< 0.001**	15 (8.8)	7 (46.7)	8 (53.3)	**0.050**
1 < 18	40 (26.5)	12 (30.0)	28 (70.0)		18 (10.5)	3 (16.7)	15 (83.3)	
18–30	15 (9.9)	10 (66.7)	5 (33.3)		89 (52.0)	45 (50.6)	44 (49.4)	
31–40	12 (7.9)	10 (83.3)	2 (16.7)		37 (21.6)	16 (43.2)	21 (56.8)	
> 40	11 (7.3)	11 (100.0)	0 (0)		12 (7.0)	8 (66.7)	4 (33.3)	
Year								
2017	31 (20.5)	12 (38.7)	19 (61.3)	0.657	26 (15.2)	12 (46.2)	14 (53.8)	1.000
2018	57 (37.7)	28 (49.1)	29 (50.9)		70 (40.9)	32 (45.7)	38 (54.3)	
2019	63 (41.7)	29 (46.0)	34 (54.0)		75 (43.9)	35 (46.7)	40 (53.8)	
Time of year								
Jan–Mar	19 (12.6)	8 (42.1)	11 (57.9)	0.419	43 (25.1)	19 (44.2)	24 (55.8)	0.360
Apr–Jun	42 (27.8)	15 (35.7)	27 (64.3)		39 (22.8)	20 (51.3)	19 (48.7)	
Jul–Sep	46 (30.5)	23 (50.0)	23 (50.0)		47 (27.5)	25 (53.2)	22 (46.8)	
Oct–Dec	44 (29.1)	23 (52.3)	21 (47.7)		42 (24.6)	15 (35.7)	27 (64.3)	
Religion								
Hindu	120 (79.5)	51 (42.5)	69 (57.5)	0.061	120 (70.2)	51 (42.5)	69 (57.5)	0.239
Muslim	24 (15.9)	16 (66.7)	8 (33.3)		46 (26.9)	26 (56.5)	20 (43.5)	
Others	7 (4.6)	2 (28.6)	5 (71.4)		5 (2.9)	2 (40.0)	3 (60.0)	
States								
Uttarakhand	102 (67.5)	42 (41.2)	60 (58.8)	0.208	108 (63.2)	42 (38.9)	66 (61.1)	**0.041**
West UP	42 (27.8)	24 (57.1)	18 (42.9)		56 (32.7)	33 (58.9)	23 (44.1)	
Others	7 (4.6)	3 (42.9)	4 (57.1)		7 (4.1)	4 (57.1)	3 (42.9)	
Hills/Plains								
Hills	55 (36.4)	18 (32.7)	37 (67.3)	**0.018**	51 (29.8)	17 (33.3)	34 (66.7)	**0.030**
Plains	96 (63.6)	51 (53.1)	45 (46.9)		120 (70.2)	62 (51.7)	58 (48.3)	
Rural/Urban								
Rural	84 (55.6)	32 (38.1)	52 (61.9)	**0.048**	82 (48.0)	34 (41.5)	48 (58.5)	0.149
Urban	67 (44.4)	37 (55.2)	30 (44.8)		89 (52.0)	45 (50.6)	44 (49.4)	
Wards								
NICU	9 (6.0)	7 (77.8)	2 (22.2)	**< 0.001**	2 (1.2)	1 (50.0)	1 (50.0)	0.496
Paed	74 (49.0)	25 (33.8)	49 (66.20		14 (8.2)	6 (42.9)	8 (57.1)	
O&G	—	—	—		120 (70.2)	57 (47.5)	63 (52.5)	
GM	14 (9.3)	10 (71.4)	4 (28.6)		14 (8.2)	4 (40.0)	6 (60.0)	
Optha	16 (10.6)	6 (37.5)	10 (62.5)		10 (5.8)	6 (42.9)	8 (57.1)	
Derma	14 (9.3)	13 (92.9)	1 (7.1)		4 (2.3)	2 (50)	2 (50)	
Others	24 (15.9)	8 (33.3)	16 (66.7)		7 (4.1)	5 (71.4)	2 (28.6)	

*Note:* Total *N*: 322. Bold values (*p* values) are for “statistically significant values” i.e., *p* values less than or equal to 0.05.

Abbreviations: Derma, dermatology; GM, general medicine; Neg, negative; NICU, neonatal intensive care unit; O&G, obstetrics and gynecology; Optha, opthalmology; Paed, paediatrics; Pos, positive; sig.: signi,cance (2-sided *p* value).

^1^For age categories, the lower limit is the completed given age (in years) and the higher limit is the completed given age (in years) up to one day less than the succeeding year.

^2^Percentage is calculated by taking the total number of cases (151 for males and 171 for females) as the denominator.

^3^Percentage calculated taking “N” (number of cases) in that particular group as the denominator.

^4^2-sided *p* value calculated by Fisher's exact test.

**Table 3:** Serological evaluation of antiherpes simplex virus 1 and 2 antibodies (IgM and IgG).

		Males (*N* = 151)	Females (*N* = 171)	*p* value2
IgM	POS *n* (%)	11 (7.3)	9 (5.3)	0.453
	95% CI1	[3.1–11.4]	[2.4–9.7]	
	Mean (AI)	0.36	0.38	
	SD	± 0.44	± 0.49	

IgG	POS *n* (%)	66 (43.7)	74 (43.3)	0.938
	95% CI1	[35.8–51.6]	[35.9–50.7]	
	Mean (AI)	1.04	1.71	
	SD	± 1.42	± 2.63	

Both IgG and IgM	POS *n* (%)	8 (5.3)	6 (3.5)	
	95% CI1	[1.7–8.9]	[1.3–7.5]	

Overall	POS *n* (%)	69 (45.7%)	79 (46.2)	0.928
	95% CI1	[37.8 - 53.6]	[38.7–53.7]	

Abbreviations: AI, antibody index; CI, confidence interval; POS, positive.

^1^Confidence interval is calculated using the normal approximation to the binomial calculation.

^2^2-sided *p* value calculated by Fisher's exact test on the positivity of IgM, IgG, and overall positivity between the two genders.

## Data Availability

The datasets in this study are available from the corresponding author on reasonable request.
